# Realist approach to community-based participatory research on a community health break-down in Japan: mechanism reasoning, knowledge and a trust partnership

**DOI:** 10.1186/s12875-023-02037-1

**Published:** 2023-03-24

**Authors:** Seiji Yamashiro, Keiichiro Kita

**Affiliations:** Department of General Medicine, Toyama University Hospital, Toyama, Japan

**Keywords:** Aging populations, Shortage of doctors, Community health break-down, Human resources development, Collaboration, Community-based participatory research, Realist approach, Context-mechanism-outcome configuration, Knowledge, Trust partnership

## Abstract

**Background:**

Our 10-year programme of community health regeneration and community-based participatory research (CBPR) was initially unknown. However, we succeeded in creating a collaboration between residents, medical staff, and administrative staff. We adopted a realist approach as an evaluation method.

**Methods:**

The realist approach evaluates a programme using a Context-Mechanism-Outcome configuration (CMOc), which is a relatively new methodology. First, the programme manager summarised the entire programme, conducted questionnaires and interviews with seven core members, and summarised each into a CMOc. The programme was evaluated with particular focus on mechanistic reasoning.

**Results:**

The number of doctors and nurses increased and residents became more active. The success factors were the acquisition of participants' knowledge and trust partnerships. In addition, it was important that the timing of the activity was good and that the participants were highly conscious.

**Conclusions:**

The 10-year CBPR was examined using a realist evaluation method. Knowledge acquisition and trust partnerships are important for reasoning mechanism.

## Background

In Japan, the birth rate is declining and the population is aging, leading to concerns about a decline in the working population, a decline in regional vitality, and an increase in demand for health and welfare services. The administrative system has been reviewed in rural areas, and the merger of cities, towns and villages has been promoted since approximately 2000.

The postgraduate medical clinical training programme began in 2004. Prior to 2004, the clinical training system for postgraduate doctors in Japan required that clinical training be conducted for two years or more after graduating from medical school, which was not compulsory. this was straightforward training by a single clinical department. Additionally, in many clinical training hospitals, training is provided in the clinical department of the university (medical office) of origin. Therefore, the number of residents receiving training through the comprehensive medical care method (super-rotation), which allows them to acquire a wide range of medical abilities, has decreased. Therefore, in 2004, two years of clinical training became compulsory to acquire the basic clinical abilities of primary care, and a clinical training system based on the matching system was started for the first time. The amount of training in clinical training hospitals other than university hospitals has increased; as a result, the number of doctors dispatched from universities has decreased at small- and medium-sized hospitals in different regions, resulting in a shortage of doctors. In particular, the shortage of doctors in small- and medium-sized hospitals in rural areas has become noticeable.

In Nanto City, Toyama Prefecture, Japan, as a result of merging of cities, towns and villages in 2005, the population was 58,140, an aging (aged 65 or over) rate of 28.5% (the national average 20.2%), the number of caregivers required was 14.9% (the national average16.7%). In addition, the consolidation of hospitals and clinics progressed, and the initial clinical training system for doctors began in 2004. However, securing doctors has become challenging. The shortage of doctors has worsened, as the number of doctors was 88 in 2005 decreased to 80 in 2012. In addition, the aging of the region has progressed, and owing to the merging of cities, towns, and villages, the cooperation of residents has weakened, causing community health breakdown in hospital medical care.

Under these circumstances, the former director of Nanto Municipal Hospital asked the first author to cooperate in preventing the collapse of regional medical care. In the first two years, we held nine seminars to explain the plight of community medicine for local residents; however, this did not lead to change as no clear methodology for action was established. Therefore, the first author considered what was necessary and investigated similar cases in other regions throughout Japan as reported in newspaper articles and books. As a result, it was found that it was important to develop human resources for medical professionals including doctors in the area and to work on a community participation system. In 2009, we started a human resources development course called ‘Meister Training Course’, and held meetings of members finishing the course every three months for the purpose of regularly exchanging information. In other words, in collaboration with the government, efforts have begun for residents, the government, and medical professionals work together. The Meister Training Course is a workshop-style course for learning and practicing healthcare. This is a community-based participatory research (CBPR) programme and a collaborative effort by universities to resolve local issues. The first author is the program manager.

The workshop was the core of this CBPR program, which was held five times over three months, with lectures and group discussions lasting two and a half hours each. In the fifth and final round, each person announced their own plans for community medical regeneration. In the lecture, they learned about four-screen thinking (awareness. change, and practice), community-based comprehensive care/community symbiotic society, and case reports of other communities. After group discussions, we formulated our own healthcare-related efforts and announced them on the final day. This course recruits approximately 50 participants every year. Participants were mainly recruited from within the region, but a very small number of participants from outside the region also participated if they wanted.

The Association for Protecting and Nurturing Community Healthcare held a meeting where Meisters gathered to listen to lectures on community medicine and comprehensive community care, and participants continuously exchanged information with each other. During the lecture, we invited lecturers who were active all over the country to learn about medicine, long-term care, welfare, and health-promotion activities. In addition, as a group activity, the doctor’s group started a training residency programme, the nurse’s group set up a study session for home-visit medical care, and the inhabitants collaborated to explore community activities.

For this year's plan, the first author (the programme manager), core members, and government officials met regularly to decide on the content, publicise it to the residents, and recruit participants. We exchanged information on our respective activities and boosted each other's morale.

The annual schedule involves holding the Meister training course over three months in the fall and then facilitating meetings to protect and nurture community medicine three times a year every three months. Meetings are held to combine group activities and government efforts. In this year’s meeting, the government acted as a coordinator, and a collaborative effort between residents, the government and medical staff was completed.

For 10 years, the number of doctors and nurses has increased; thus, the collapse of medical care has been resolved, the activities of residents have been activated, and it had become possible for residents, medical staff, and the government to cooperate. The 10-year success rate can be evaluated empirically using real numbers. However, little is known about how to evaluate what is good about the effort. Here, we introduce a methodology called the realist approach to empirical evaluation.

Only two previous studies adopted a realist approach in Japan, both of which were programme evaluations for multidisciplinary education [1, 2]. There are no Japanese reports on the use of a realist approach to CBPR programmes, as in this study. Therefore, we evaluated why a 10-year CBPR programme with annual cycle of activities continued and how it produced results using a realist approach.

## Methods

The realist approach is a theory-driven approach advocated by Pawson and Tilley. It is an evaluation methodology within the framework of context-mechanism-outcome [3, 4]. The context-mechanism-outcome configuration is presented in Table 1. This context describes in what circumstances and for whom. The mechanism is a framework for evaluating the type of intervention performed and how the outcomes were obtained. Dalkin et al. divided the mechanism into two parts: intervention (programme/intervention/resources) and reasoning (assumption/reasoning). They evaluated the palliative care Integrated Care Pathway (ICP) programme by dividing the programme mechanism into resources and reasoning. This was based on the fact that terminally ill patients with non-cancer diseases were enrolled less frequently than terminally ill patients with cancer. It turned out that the difference in anxiety in mechanism reasoning appeared in the difference in registration. They clarified the concept of mechanism through a realist evaluation. That is, M (resource) + C → M (reasoning) = O. We focus on the reasoning behind the latter mechanism [5] in Table 2.

**Table 1:** Context-mechanism-outcome configuration

Context-mechanism-outcome (CMO)	A CMO configuration is a proposition stating what it is about a programme which works for whom in what circumstances.
Context	Realist evaluators seek to understand ‘for whom and in what circumstances’ a programme works through the study of contextual condition.
Mechanism	Realist evaluators seek to understand ‘why’ a programme works through an understanding of the action of mechanisms.
Outcomes	Outcomes provide the key evidence for the realist evaluator in any recommendation to mount, monitor, modify, or mothball a programme.

**Table 2:** Mechanism components

Mechanism (Resources) + context → Mechanism (Reasoning) = Outcome	Using this formula, it became clearer whether data contributed contextually or mechanistically, could identify mechanism components (resource and reasoning), which are different depending on the contexts.

First, the first author applied our 10-year CBPR programme to the CMOc. We then emailed the questionnaire to seven core members (two community residents, two community nurses, two administrative officers, and one doctor [program deputy manager]) and interviewed them according to the questionnaire in Table 3. The interviews took 40–60 min. All participants signed a consent form before the interview began. We summarized these results using the CMOc. The interview results were anonymous and Informed consent was obtained.

**Table 3:** Questionnaire

1) Context of your activities
a. In what circumstances have you started? Why and How b. For whom
2) Mechanism of your activities
a. What worked
3) Outcome of your activities
a. What was the outcome? b. What are the effect of your activities?
4) Rating assessment
1.Very much below expected, 2. Slightly below expected, 3. Expected, 4. Slightly more than expected, 5. Much more than expected
5) In what area what did you make the most effort?
6) What was your motivation for your activities?
7) Free comment

## Results

In the two years of seminar activities before the programme, there was no increase in doctors and nurses, and no new activities took place. However, through the seminar activities, the participants came to understand the problem in the region and the residents and government became more aware of the crisis in community medicine, which led to the next 10-year programme.

**Fig. 1:**
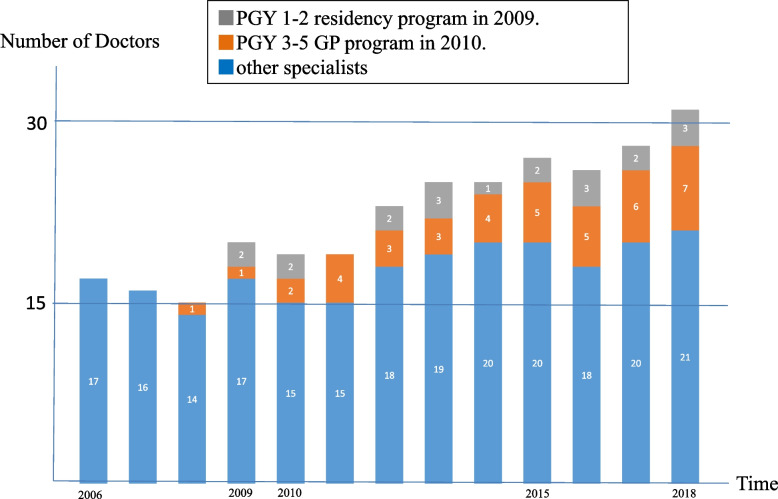
Number of doctors has increased

**Fig. 2:**
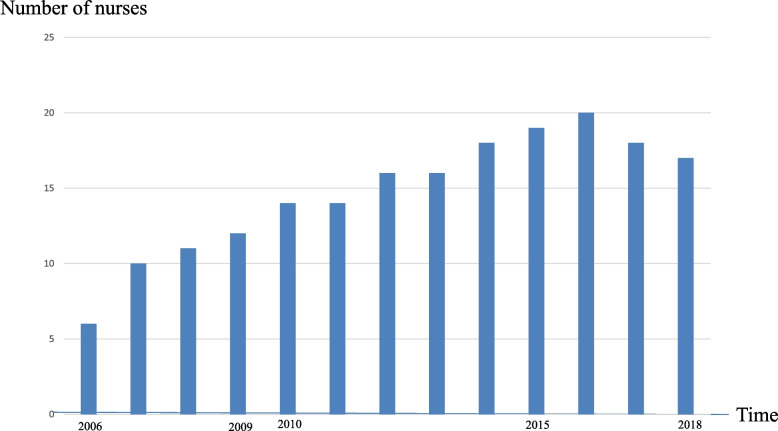
Number of community nurses has increased

**Fig. 3:**
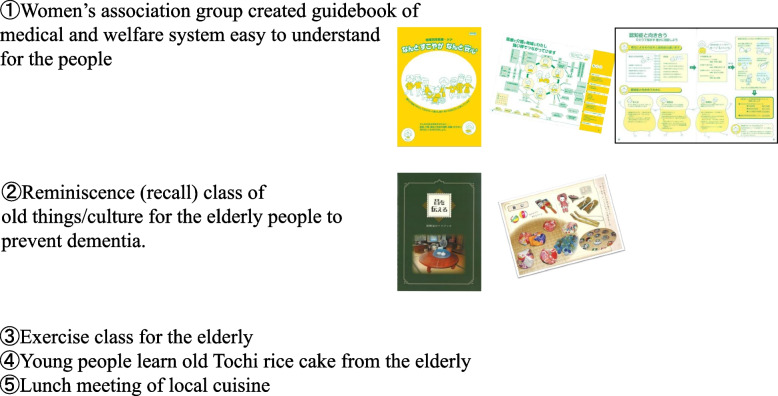
Residents became more active Fig. 4 Our Program of CBPR

During 10-year programme, 428 people completed the training course. In addition, we held 30 meetings to protect and nurture community medicine. During 10-year period, the postgraduate training programme and GP specialty training programmes were launched at Nanto Municipal Hospital, and the number of doctors doubled from 15 to 30 (Fig. 1). In addition, community nurses conducted study sessions, and the number of nurses increased from 11 to 18 (Fig. 2). Resident groups also became active (Fig. 3).

**Fig. 4:**
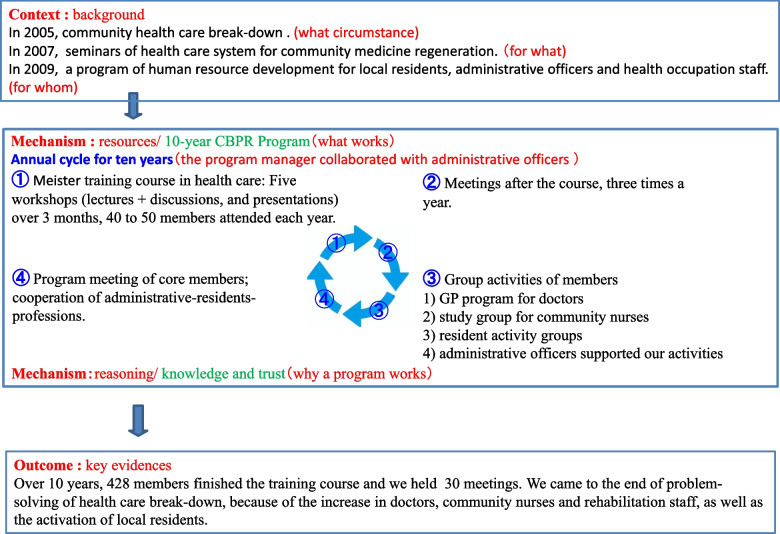
Our Program of CBPR

As a program manager, the author summarized the program in the CMOc, as shown in Fig. 4. and Table 4. Knowledge and trust were mentioned as reasoning mechanisms that persisted for 10 years. In term of knowledge, four-screen thinking and a community-integrated care system in an aging society were used as methods for changing consciousness during the training course. The group work during the course improved communication and created trust partnerships.

**Table 4:** CMOc of programme manager

Mechanism (reasoning)(Purpose)	Knowledge: Acquisition of methodology of awareness change (four-screen thinking) for sustainable problem solving and learning the community-integrated care system Trust: Good communication among participants.
Outcome (key evidences)	Over 10 years, 428 members finished the training course and we held the 30 meetings. During this programme, we started a residency programme for GPs at Nanto Municipal Hospital, a study group for community nurses and rehabilitation staff and an activity group for local residents. We resolved the problem of healthcare breakdown, because of the increase in doctors, community nurses, as well as the aid of local residents.

The CMOc values of the core members was summarized in Table 5. Local residents, especially women's associations, said that this programme started at a good timing for starting activities as it was around the time the Union Women's Association was formed after the merging of cities, towns and villages, and was looking for new activities. There was also anxiety about the aging of the region, and this was an opportunity to think about countermeasures. Community nurses organised study sessions, such as physical assessments to promote home care. Work and community love were especially strong among nurses. A doctor who was the director of Nanto Municipal Hospital at the time served as the deputy manager for this programme. To increase the number of doctors in the hospital, we set up residency and GP training programmes and made efforts to secure doctors. Owing to further efforts to improve the quality of hospitals and academics, the number of doctors has increased. Government officials acted as liaisons and coordinators for the programme, supporting residents and healthcare professionals. The relationship between trust among the core members who supported the program was strong.

**Table 5:** CMOc of core members

1. Community residents	
Context	Established women’s association. Anxiety regarding community health for aged people. Anxiety regarding weakening of human relations in the community.
Mechanism (intervention/resources)	Set up a community health study group creating a guidebook for community healthcare. Made a place to stay and communicate for community members.
Mechanism (reasoning)(Purpose)	Good timing due to the establishment of women’s association and working on solving local issues. Anxiety about super-aging society, from worry to practice. Anxiety stimulated efforts to deepen and spread connections with people.
Outcome	Many activities began: Communication among residents, recognition of community health, recognition of residents' role in community-integrated care, and helping with community medicine. Active interactions with people.
2. Community nurses	
Context	Head nurse responsible for home care. Need quality improvement of home care. Participated in community-integrated care system.
Mechanism (intervention/resources)	Set up a study group. Made a vision and quality improvement for community nurses.
Mechanism (reasoning)(Purpose)	Occupational love by head nurse for colleagues and community members. Good opportunities for education. Community nursed received a good evaluation.
Outcome	Increase the number of nurses and home care patients. Became top-level home care nurse station in the prefecture. Improvement of the quality of care for patients.
3. Doctor (program deputy manager)	
Context	Anxiety as a head doctor in a hospital due to a decreasing number of doctors. Community health challenges for patients and hospital stuffs.
Mechanism (intervention/resources)	Started a residency programme for GPs in the community hospital. Helping home care nurses. Participated in community-integrated care.
Mechanism (reasoning)(Purpose)	This programme began during a difficult time for the hospitals. Good motivation for the programme and efforts to improve the hospital’s quality of care and academic level.
Outcome	Increase in the number of doctors and nurses at the hospital. Improved quality of care for home care and community-integrated care.
4. Administrative officers	
Context	Needed solutions for community issues.
Mechanism (intervention/resources)	Support from programme for public relations, reports, and distribution.
Mechanism (reasoning)(Purpose)	Support from programmes and mutual understanding among community members and medical professionals.
Outcome	Established horizontal connections among city hall departments.

## Discussion

In the evaluation of our program's mechanism, it was important that its purpose was clear, and that community residents participated. Resident participation and administrative support were key to continuing the programme. At the beginning, we consulted with the person in charge of the government programme and encouraged the participation of resident representatives and medical personnel who could become leaders. We then gradually expanded the participant base. Thanks to the support of the government, residents’ participation has increased, and the awareness of the residents of their own affairs also increased. From the Meister training course to an association that protects and nurtures community medicine, each group in our programme has become active and cooperative with other groups.

It seems that the participants produced results by learning the methodology of consciousness change and its practice via the four-screen thinking method (knowledge of new actions) and communicating well with each other (trust in collaboration). Furthermore, from CMO1 to CMO4, the core members of each group became stuck in their respective activities, and this programme started when they were looking for a solution, so it was a good time for the development of the activities. It is also said that love for work and community and empathy for learning together became stronger. Therefore, knowledge and trust partnership were the key reasoning factors in our program.

In Japan, the two studies on the realist evaluation of the multidisciplinary education programmes found that observational learning was the key mechanism for medical students, and professional role-play was the key for multidisciplinary education in primary care.

Many studies have used realist evaluation in programme which were social, practical and complex and demonstrated the key points of various progarmmes. Pawson et al. said that as a result of analysing the programme contents of social activities with CMO, knowledge is brought to policymakers and the programme succeeds [3]. Belle et al. wrote about the complexity of health policies and programmes, and how a realist evaluation can reveal what will bring results [6]. In their qualitative evaluation of a clinical faculty mentorship programme, McDaniel et al. adopted a realistic approach and found that opportunities to connect, share ideas and strategies, and self-reflect were useful in achieving meaningful outcomes [7]. Sriranjan et al. studied the diagnosis of postpartum depression in general practice using case vignettes using a realistic approach. The personal and clinical experiences of GPs and effective communication channels with other primary care professionals are significant mechanisms [8]. Gordon et al. reported that investment, trust between practitioners, wrapping around care, and access to specialist care were effective factors in their realist evaluation of optimal healthcare delivery to care homes [9]. Francis-Coad et al. evaluated a fall prevention community in a residential aged care setting using a realist approach. The evaluation was divided into three levels, and members, facilities, and organizations were evaluated through survey, audits, observations, and semi-structured interviews. Participant cooperation and knowledge acquisition led to falls prevention, and the facilities and organizations were members. They reported that investing time was the key to success [10]. Bertotti et al. evaluated social prescribing using a realist evaluation. After interviewing at three levels (GP, social prescribing coordinator, and community/statistics organisations), the key mechanism was found to be the social interaction with the patient at each level, with increasing trust as the primary point [11]. Calo et al. evaluated the effect of a community-based music intervention on young people, especially those who are disadvantaged, using a realist evaluation. It has been reported that the achievement of trust and connectedness improves young people’s self-confidence and well-being [12].

Jagosh et al. evaluated CBPR using a realist evaluation and reported that its success lay in the fact that partnership synergy, trust relationships, and related ripple effects were derived from trust building, self-empowerment, and knowledge production in mechanism reasoning. The long-term effects were connected with CMO1 → CMO2 → CMO3, which brought about the development of CBPR [13].

Our program has developed from a two-year seminar to a ten-year programme, and a new development is currently underway. Our programme is not an extension of the three CBPRs. However, when the programme was divided into three phases, it followed a similar pattern. In other words, in mechanism reasoning, we realized that trust partnership and knowledge were important for self-empowerment.

## Limitations

We adopted a realist approach for the first time and interviewed core members after the end of the 10-year programme. Although the core members had a strong relationship with trust, we did not interview other participants. However, further investigation is required. In addition, we believe that it is necessary to deepen our understanding of the realist approach and compare it with other CBPR evaluations in Japan.

## Conclusion

We evaluated a 10-year CBPR programme using realist approaches. Questionnaire surveys and interviews with programme managers and core members revealed that the mechanism reasoning was a knowledge and trust partnership with a clear common purpose.

## Data Availability

The datasets generated and analyzed during the current study are available from the corresponding author upon reasonable request.
